# Mutual Coupling Reduction in MIMO DRA through Metamaterials

**DOI:** 10.3390/s23187720

**Published:** 2023-09-07

**Authors:** Muhammad Sabir Khan, Shahid Khan, Owais Khan, Sajid Aqeel, Neelam Gohar, Mariana Dalarsson

**Affiliations:** 1 Department of Electrical and Computer Engineering, COMSATS University Islamabad, Abbottabad-Campus, Abbottabad 22060, Pakistan; sabir5775@gmail.com (M.S.K.); shahid@cuiatd.edu.pk (S.K.); engrowais615@gmail.com (O.K.); sajidaqeel@cuiatd.edu.pk (S.A.); 2 Department of Computer Science, Shaheed Benazir Bhutto Women University, Peshawar 25000, Pakistan; neelam.gohar@sbbwu.edu.pk; 3 School of Electrical Engineering and Computer Science, KTH Royal Institute of Technology, SE 100-44 Stockholm, Sweden

**Keywords:** metamaterial, split-ring resonator, dielectric resonator antenna, mutual coupling, MIMO

## Abstract

A single negative metamaterial structure with hexagonal split-ring resonators (H-SRRs) is inserted within a two-port multiple-input multiple-output (MIMO) dielectric resonator antenna (DRA) in order to achieve a reduction of mutual coupling between closed multiple antenna elements. Between closed, tightly coupled, high-profile antenna elements, the single negative magnetic inclusions (H-SRRs) are embedded. By incorporating magnetic structures within antenna elements, the mutual coupling is significantly diminished. Mutual coupling reduction is attained by inserting an array of hexagonal split-ring resonators between the inter-spacing elements. An operative approach for the reduction of the mutual coupling between two × two MIMO DRAs initially operating at 5.2-GHz band is provided. To make the simulated design replica of the fabricated prototype, an air gap is introduced between the substrate, DRs, and H-SSRs. The addition of the air gap shifts the simulated results to 5.9 GHz, which closely resembles the measured values. The mutual coupling reduction is realized by integrating a meta-surface amid the two × two MIMO DRAs, which are settled in the H-plane. The meta-surface embraces an array of hexagonal split-ring resonator (H-SRR) cells that are unified along the E-plane. The H-SRR structure is designed to offer band-stop functionality within the antenna bandwidth. The proposed design has an overall dimension of 40 × 58.3 × 4.75 mm^3^ (1.5λ × 1.02λ × 0.079λ). By stacking the DRA with a one *×* three array of H-SRR unit cells, a 30 dB reduction in the mutual coupling level is attained without compromising on the antenna performance. The corresponding mutual impedance of the MIMO DRA is better than 30 dB over 5.9–6.1 GHz operating bandwidth. The proposed design has a DG of 10 db, ECC < 0.02, CCL < 0.02 bits/s/Hz, and an MEG of 0 dB. The overall design has a promising performance, which shows its suitability for the target wireless application.

## 1. Introduction

During the last decade, with the development of communication systems, the demand of the capacity and data rates for internet and other services (texting, browsing, streaming, global positing system, etc.) has highly increased. This technological development has considerably increased the number of subscribers. To fulfill the new requirements, the concept of multiple antennas (MIMO) was proposed. The main challenge of the multiple antennas device/system is the size constraint. Electronics system design engineers are therefore focusing on the miniaturized transmitting/receiving (Tx/Rx) devices, while maintaining a high data rate, enhanced channel capacity, and better coverage of the signal [1]. There is a growing demand for miniaturized multiple antennas devices. Based on theoretical information, multiple antenna elements need to be placed at a mutual distance of λ/2 for appropriate coupling and low correlation between the elements. It is important to note that the performance of a multiple antennas system is deteriorated when the elements are too closely placed, due to an increase in the mutual coupling (MC). For a MIMO antenna system, the performance is severely affected by the electromagnetic interaction (mutual coupling) between the antenna elements. Mutual coupling is the electromagnetic interaction between the antenna elements which has adverse effects on multiple antennas (MIMO) performance. The closely placed antennas generate high coupling, which deteriorates the antenna system performance [2]. Mutual coupling effects the radiation pattern, input impedance, signal-to-noise ratio (SNR), feed impedance, reflection loss, and correlation of signals. The reduction of electromagnetic coupling within comprising elements is a challenging design task.

In [3,4,5,6,7,8,9,10,11,12,13,14,15], several methods have been debated in the literature on how to reduce the effects of MC. However, these methods are not compatible for all types of multiple antennas. Apart from these conventional techniques, recently metamaterials have been discussed as an alternative for the mutual coupling reduction. Metamaterials are artificially manufactured materials that exhibit unique properties normally not found in naturally occurring materials [3]. In general, the artificial materials that have undesirable properties (negative permittivity ε or negative permeability μ or both negative permittivity and permeability) are referred to as metamaterials [4,5]. Using metamaterials blocks the current flow between the antenna elements and ports. This highly enhances the port isolation and reduces mutual coupling. Enhanced isolation is helpful to further squeeze the separating distance between the antenna elements without increasing the mutual coupling [6]. For this reason, several configurations of metamaterials have been investigated, like electromagnetic band gap (EBG) [8,9], high impedance surfaces (HIS) [10], complementary split-ring resonator (CSRR) [11,12], and split-ring resonator (SRR) [13,14]. In all of them, CSRR and SRR configurations offer higher reduction of MC than others [15]. A decoupling layer based on artificial magnetic materials (AMMs) is established to alleviate the electromagnetic interaction effects encountered between high profile antenna systems [15,16]. The designed decoupling layer, which consists of an array of uniquely shaped SRRs, is placed between highly coupled antenna elements. The decoupling layer not only provides high mutual coupling suppression, but it also maintains good impedance matching. This is achieved with a very small separation distance between the elements (less than λ/2), where λ is the operating wavelength of the antenna elements [16]. Such structures are simple in design, compact in size, and easy to integrate in multiple antenna elements. The detailed studies related to unique (complementary) split-ring and common split-ring resonators have been discussed in the literature [11,12,17,18,19,20,21]. According to all discussions, they are essential for mutual coupling reduction and maintaining high performance in MIMO antenna systems. One effective technique is discussed to improve the isolation between two MIMO DRAs working on 60 GHz for MIMO application [17]. This is achieved by using a meta-element unique SRR that is designed to offer band stop working over the resonance frequency range. Using the proposed technique, the reduction of mutual coupling between the antennas is improved 20 dB [18]. In this research, isolation is enhanced between closely spaced monopole MIMO antennas. The metamaterials (SRRs) built in this research have a negative strong magnetic response. The magnetic enclosures present their efficiency in terms of isolation enhancement and their protecting efficiency in overpowering the displacement current. Thus, the MNG (μ-negative) slab is an efficient magnetic protecting wall that can be useful in multiple antenna (MIMO) applications [19]. The results show that more than 20 dB isolation between antennas was attained. Port isolation without an MNG slab of 7 dB is changed using an MNG slab of 27 dB.

In [20], a spiral split-ring resonator (spiral SRR) is offered as a beneficial solution for isolation enhancement in a microstrip array antenna that contains two elements. The parameters of design spacing, number of rows, width, and length were studied. To validate the performance of the offered filtering element and to progress the reduction of mutual coupling among the closely spaced antenna elements, complementary split-ring resonator designs are implemented between the elements. The benefit of the offered structure is its small size (nearly λ/10) and its ease of fabrication compared to other metamaterial designs [21].

Mutual coupling highly depends on the spacing between the antenna elements. Closely spaced antenna elements offer high coupling with reduced port isolation. This causes unwanted effects on antenna features like reduced ECC and low DG, TARC, and MEG. Contrary to closely spaced antenna elements, increasing the inter elements distance reduces the mutual coupling, but it causes an enlarged size and affects the radiation pattern. Within a compact device, reduction in mutual coupling in MIMO antennas is a major challenge. As the proposed design is working at a lower frequency, handling the mutual coupling at this range of frequencies is even more challenging. We need techniques which are helpful in reducing the mutual coupling while maintaining a compact size and high performance. Thorough investigation of multiple techniques reveals that the deployment of metamaterials (CSSRs and SSRs) among the antenna elements is the ultimate solution to this issue. These uniquely structured materials have the potential to highly reduce the mutual coupling while maintaining a good overall performance [22].

The main objective of this work is to combine H-SRRs for first time with RDRA MIMO antenna elements for mutual coupling reduction. H-SSRs offer wideband response with a compact size. Previously, this technique was combined with cylindrical DRAs. It is well known that RDRA has different operating modes and shape than the cylindrical DRA. Thus, integrating H-SRR with RDRA is a real challenge. Careful design and optimization have enabled a successful deployment which results in a significant reduction in mutual coupling (from 6 dB to 30 dB).

The rest of the paper is organized as follows: Section 2 discusses the proposed design, Section 3 discusses the design evaluation steps, and Section 4 discusses parametric studies. Section 5 discusses the simulated and measured results, Section 6 gives details of the MIMO performance of the proposed design, and Section 7 concludes the paper.

## 2. Design of Proposed MIMO DRA

**Figure 1:**
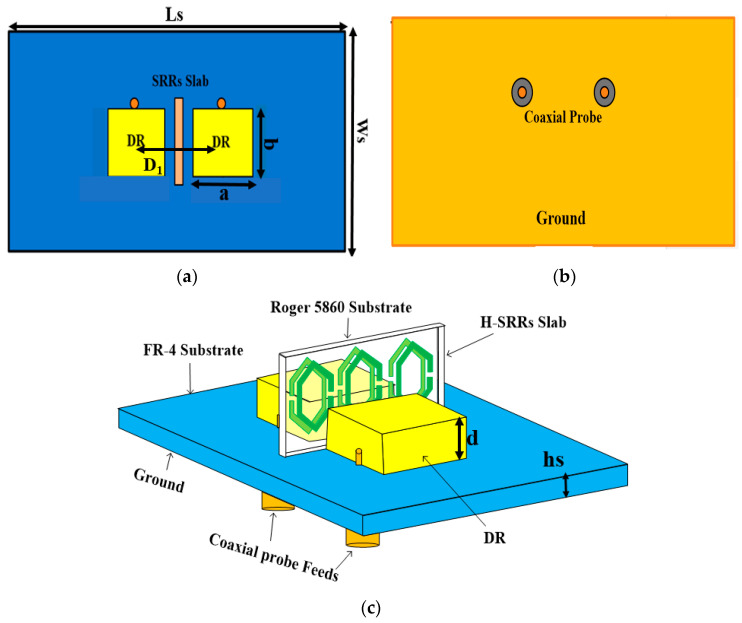
The proposed MIMO-DRA geometry: (**a**) top view, (**b**) bottom view, (**c**) perspective view.

Figure 1a–c indicate the proposed antenna top, bottom, and trimetric configuration. The proposed two-port MIMO DRA is positioned on a low-cost FR4 substrate material having a 4.3 dielectric constant and 0.025 loss tangent. The substrate has total dimensions of Ls × Ws × hs, where Ls, Ws, and hs are the length, width, and height of the substrate, respectively. The proposed MIMO DRA consists of two identical rectangular DRs, which are positioned on the top surface of the substrate. Each DR is made of ceramic material having a 30 dielectric constant. The DRs have dimensions of a × b × d. Here a, b, and d are the length, width, and height of the DR. The DRs are placed in a linear arrangement on the substrate. d_1_ is the center-to-center distance between the DRs. Each DR of the proposed design are excited by a coaxial probe feed, which is placed at the bottom of the ground plane. For the isolation enhancement, a metamaterial structure with three pairs of hexagonal-shaped split-ring resonator (H-SRR) is introduced between the DRs. The detailed dimensions of the proposed design are listed in Table 1.

## 3. Evaluation of Proposed Design

**Figure 2:**
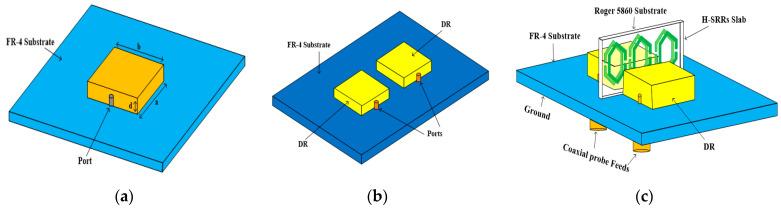
Design steps of proposed design: (**a**) single rectangular DRA, (**b**) MIMO DRA without H-SRR, (**c**) proposed design.

**Figure 3:**
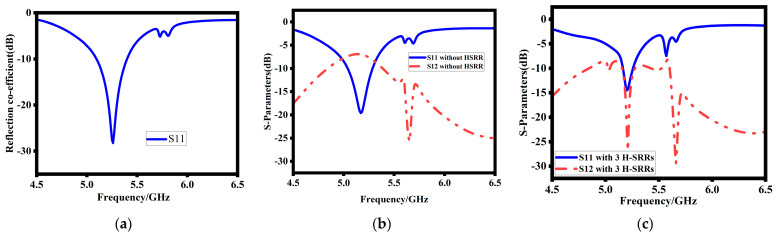
(**a**) Reflection coefficient of (**a**) single DRA, (**b**) MIMO DRA without H-SRR, (**c**) proposed design.

The evaluation process of the MIMO DRA is depicted in Figure 2 and its corresponding reflection coefficients are shown in Figure 3. In the first step, a single rectangular DRA is designed and excited using coaxial probe feeding as shown in Figure 2a. The rectangular dielectric resonator (RDR) frequency is dependent upon their dimensions (length, breadth, height). The resonance frequency of the RDRAs is given by [1].

(1) $$f_{r}=\frac{1}{2\pi \sqrt{\epsilon_{o}\mu_{o}\epsilon_{r}}}\sqrt{k_{x}^{2}+k_{y}^{2}+k_{z}^{2}}$$

where

(2) $$k_{x}^{2}+k_{y}^{2}+k_{z}^{2}=\epsilon_{r}k_{o}^{2}$$

In (2), $k_{x},k_{y},k_{z}$ are wave numbers in three Cartesian directions given by Equations (3)–(5), $\epsilon_{r}$ is the relative permittivity of the DR material, and $k_{0}\;$is a free-space wave number, while $\mu_{o}$ and $\epsilon_{o}\;$are the vacuum permeability and permittivity, respectively.

(3) $$k_{x}^{}=\frac{m\pi }{a}$$

(4) $$k_{y}^{}=\frac{n\pi }{b}$$

(5) $$k_{z}^{}=\frac{p\pi }{d}$$

In this case, the DR resonates at 5.2 GHz with good impedance bandwidth and matching. The simulated S11 are depicted in Figure 3a. In the second step, the design is modified into two elements of MIMO (multiple-input multiple-output) configuration. In the MIMO DRA, the same single unit cell is replicated with a 14.5 mm center-to-center distance between them as shown in Figure 2b. The corresponding reflection coefficient of the MIMO DRA is depicted in Figure 3b. Finally, to enhance the decoupling and improve isolation between the MIMO DRAs, step 3 introduces a metamaterial structure with an H-SRR (hexagonal split-ring resonator) shield between the antenna elements, as shown in Figure 2c. The introduction of metamaterial structure highly improves the isolation between the MIMO elements, as shown in Figure 3c.

### SRR Configuration

**Figure 4:**
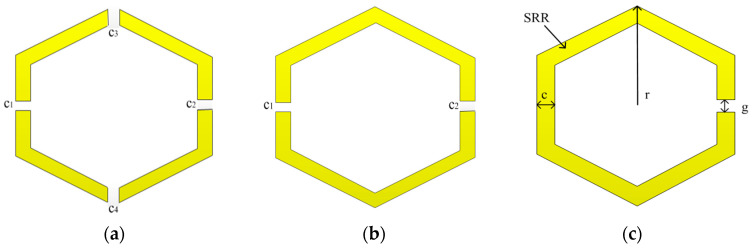
Different SRR design steps: (**a**) four-splits, (**b**) two-splits, (**c**) single-split.

**Figure 5:**
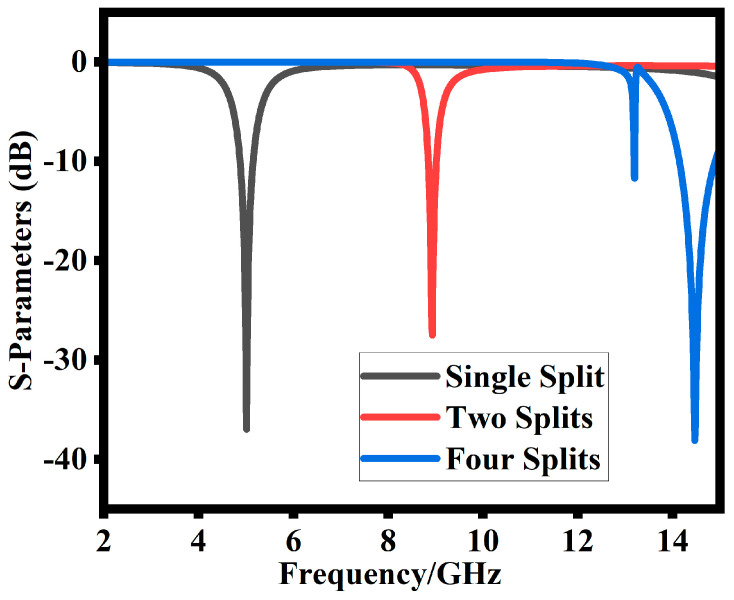
Impact of changing number of SRR splits on transmission coefficient (S12).

The unique characteristics of a metamaterial shield are employed in the proposed MIMO DRA presented in this section. The metamaterial structure comprises a unique unit cell (SRR) having dimensions “r (3.9 mm)”, “g (0.5 mm)”, and “c (0.6 mm)”, where r is the radius, g is the split width, and c is the SRR width, as shown in Figure 4. The inductance and capacitance of SRRs are created by the conductive components and ring gap, respectively. Initially, a single ring resonator (SRR) with four splits is designed, which is shown in Figure 4. The SRR reflection coefficient response as shown in Figure 5a is above 14 GHz, which is very high compared to the DR resonance frequency. Reducing the splits from four to two shifts the reflection coefficient to a lower frequency. At this stage, as shown in Figure 5b, the reflection coefficient shifts to 9 GHz, which is still higher as compared to the DR resonance frequency. Reducing the SRR split to one, as given in Figure 4c, shifts the reflection coefficient to the desired value of the DR resonance frequency. As shown in Figure 5, the reflection coefficient at this stage moves to the desired 5.2 GHz target value.

## 4. Parametric Analysis

To achieve the optimum choice of H-SRR with improved results for the proposed design, a parametric analysis is carried out on important parameters of the SRR. Different parameters of the SRR have different effects on the performance of the proposed design. These key parameters include the width of the splits and the width of the metal of the inner sides and outer sides.

**Figure 6:**
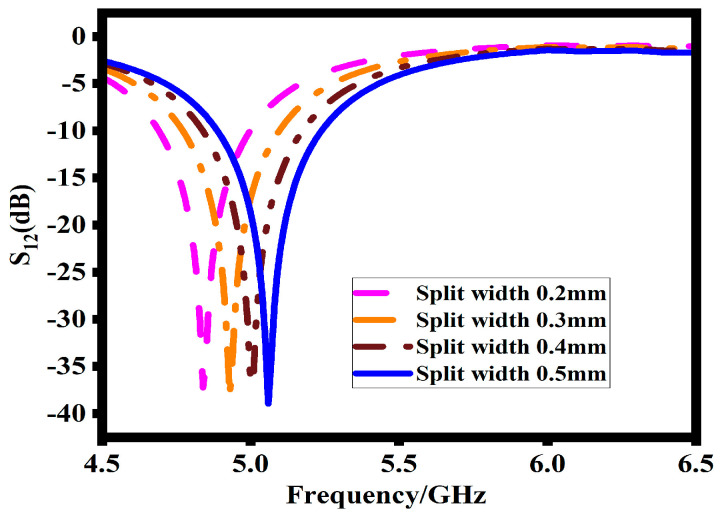
Effects of split width on transmission coefficient (S12).

As shown in Figure 6, the effect of the split width plays a crucial role in designing the SRR. The results clearly explain that increasing the split width g shifts the impedance bandwidth to a higher spectrum. This shift is described by Equation (6) for the capacitance, which describes the behavior of the split width g.

(6) $$C=\frac{\varepsilon A}{d}\;$$

Increasing the gap split decreases the capacitance, which in response increases the resonance frequency. Based on the return loss response, the width of the split is attained at 0.5 mm.

**Figure 7:**
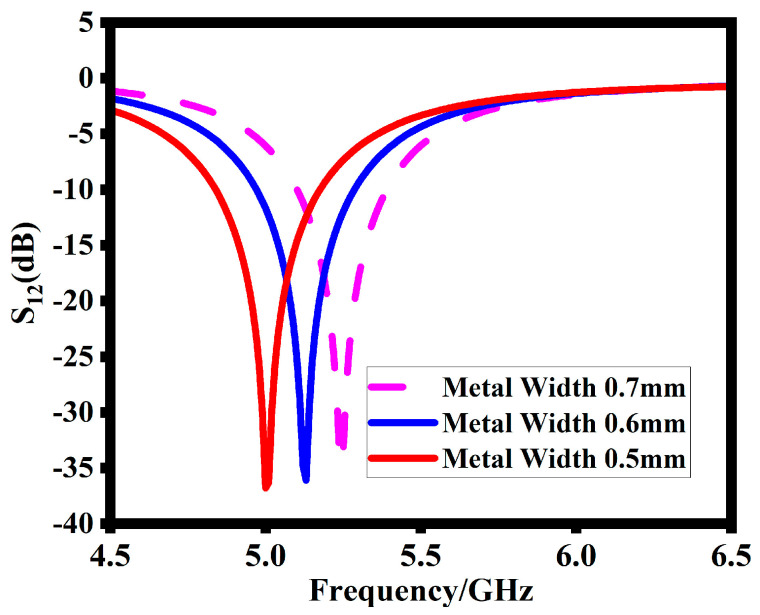
Effect of SRR strip width on transmission coefficient (S12).

The width of the SRR also plays an important role in attaining the desired resonance frequency. As shown in Figure 7, decreasing the metal width shifts the operating band to a lower range. At the metal width of 0.6 mm, the SRR resonates at 5.2 GHz. Thus, 0.6 mm is the final value of the SRR metal width for the desired resonance frequency.

## 5. Simulated and Measured Results

In this section, we present the final simulated and measured results of the proposed design, providing a comprehensive evaluation of its performance. The key parameters analyzed include reflection coefficients, mutual impedances, gain, and radiation patterns across all operating bands. The mutual impedances between the antenna ports are also examined to evaluate the level of interference and coupling between the elements.

### 5.1. Experimental Results

**Figure 8:**
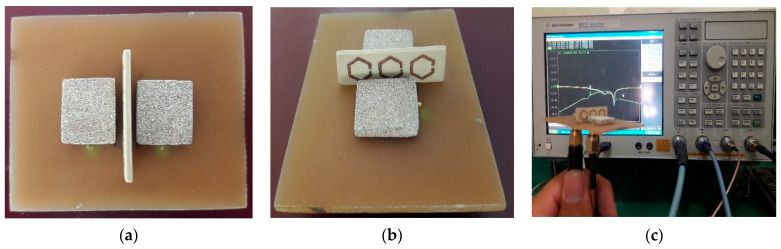
Prototype photograph of proposed MIMO-DRA with H-SRR: (**a**) top view, (**b**) side view, (**c**) measurement setup.

The proposed MIMO-DRA, integrated with a one × three array of an H-SRR unit cell, is fabricated and its performance is measured. The prototype photograph of the proposed MIMO-DRA with H-SRR is depicted in Figure 8. Figure 8a shows the top view of the fabricated prototype. Figure 8b shows the 3D view of the proposed design. An air gap is visible between the DRs, H-SSR, and substrate. Figure 8c shows the reflection coefficients’ measurement setup. The prototype is properly connected to a Vector Network Analyzer (VNA) for reflection coefficient measurement.

### 5.2. Reflection Coefficients

**Figure 9:**
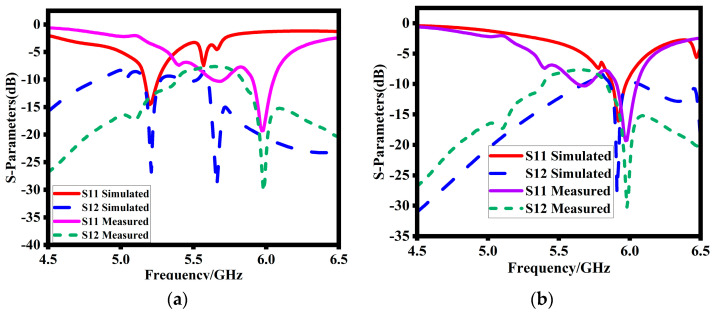
Simulated reflection coefficients and mutual impedances (**a**) without air gap and (**b**) with air gap.

Figure 9 demonstrates the analysis of the reflection coefficients of the proposed design via experiments and simulations. Figure 9a shows that the simulated design resonates at 5.2 GHz with −29 dB corresponding port isolation. Surprisingly, as shown in Figure 9b, the measure reflection coefficients are highly shifted to a higher range and resonate at 5.9 GHz with −31 dB corresponding port isolation. This right shift in the operating band is due to the existence of an air gap between the DR, H-SSR, and substrate. When this air gap is incorporated in the simulated design, the simulated results shift to a higher spectrum as well. At this stage, the simulated are in close agreement. Both the simulated and measured results are around 5.9 GHz. The minor discrepancies are due to the surrounding noise, inaccurate fabrication, and loose connections.

### 5.3. Simulated and Measured Gain

**Figure 10:**
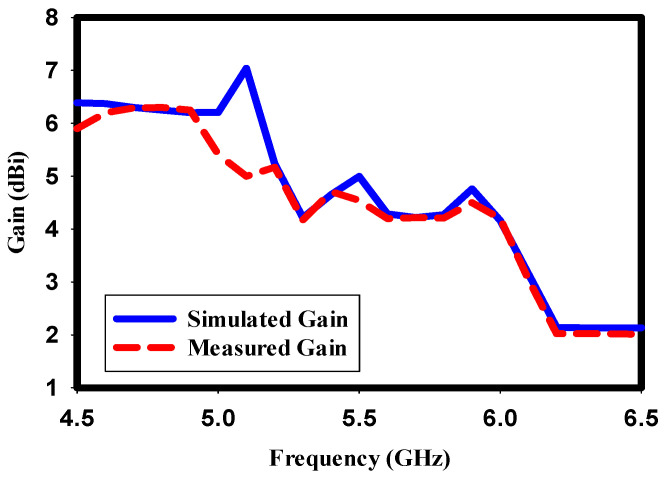
Simulated and measured gain of the proposed design.

The gain characteristic is a vital indicator of an antenna’s performance and its ability to radiate and transmit power effectively. The gain of the proposed design across the operating bands is evaluated through simulation and measurement, as depicted in Figure 10. Overall, the measured gain values closely agree with the simulated results at mid-range frequencies. However, a minor deviation is observed at the lower and higher operating bands. This discrepancy may be attributed to various factors, including fabrication tolerances and measurement uncertainties. Despite this slight deviation, the general agreement between the simulated and measured gain demonstrates the effectiveness of the design in achieving desirable radiation characteristics. The design has a peak gain of 5 dBi at the target resonance frequency.

### 5.4. Surface Current Distribution

**Figure 11:**
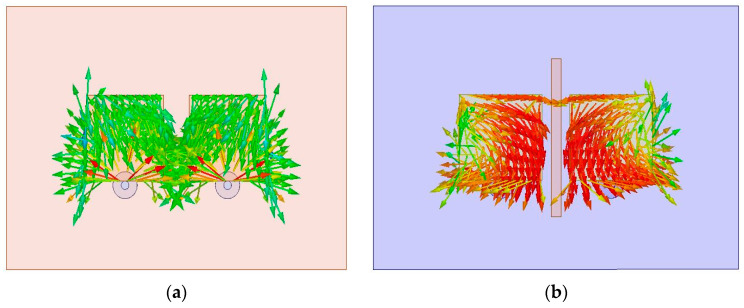
(**a**) Current distribution of the proposed design at 5.8 GHz before decoupling technique; (**b**) current distribution of the proposed design at 5.8 GHz after adding decoupling technique.

Surface current distribution is helpful in explaining the overall behavior of the proposed design. Figure 11a,b show the current distribution of the proposed design before and after the decoupling technique. Figure 11a shows that in the absence of hexagonal SRRs, there is a current flow between the ports and MIMO elements. This current flow reduces the port isolation and causes an increase in the mutual coupling. Figure 7b shows that adding the decoupling technique highly reduces the current flow between the MIMO DRA elements. This current reduction between the MIMO elements causes an enhancement in port isolation, thus causing a decreased mutual impedance. The same response is also clear from the S-parameter of the proposed design at 5.8 GHz.

### 5.5. Far Field Radiation Characteristics

**Figure 12:**
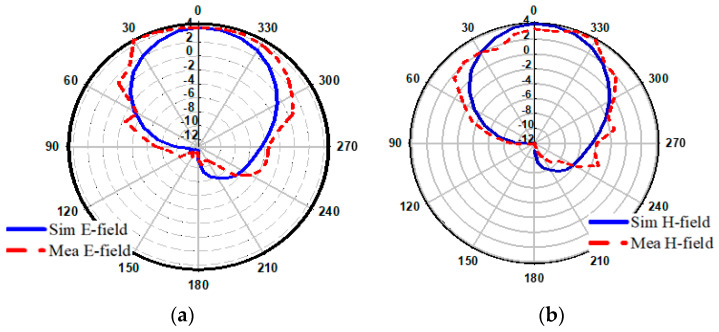
2D Simulated and measured radiation pattern of proposed design at 5.9 GHz; (**a**) E-field and (**b**) H-field, respectively.

Figure 12a,b present the electric (E) and magnetic (H) far-field radiation patterns of the proposed design at the target resonance frequency. The far-field patterns were measured in an anechoic chamber. The radiation patterns were obtained by activating Port 1 while terminating Port 2 with a 50-ohm load. The simulated and measured radiation patterns for both ports exhibit a close agreement, displaying an omni-directional nature. The radiation patterns repeat the same pattern for each port, indicating consistent performance across the operating bands. The peak gain for both ports varies between 2 dBi and 6.08 dBi, providing a measure of the antenna’s radiated power in different directions. It should be noted that minor discrepancies between the simulated and measured radiation patterns at 5.9 GHz can be attributed to non-ideal measuring environments and fabrication errors. These factors can introduce slight variations in the antenna’s performance at specific frequencies.

## 6. MIMO Parameters

To assess the MIMO and diversity performance of the proposed antenna, key parameters such as the envelope correlation coefficient (ECC) and diversity gain are evaluated.

### 6.1. Envelop Correlation Coefficient

**Figure 13:**
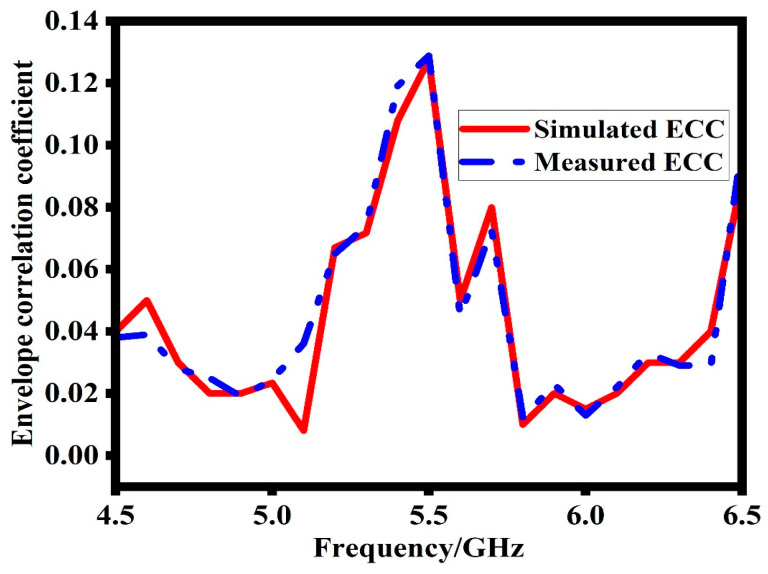
Simulated and measured envelope correlation coefficient of the proposed design.

The ECC is a crucial metric in MIMO antenna systems as it quantifies the correlation or isolation between the different branches of communication. In this study, the ECC is computed using the radiation pattern of the proposed MIMO antenna and can be expressed using Equation (7). The measured pattern is noticed at three frequencies (5.7 GHz, 5.8 GHz, and 5.9 GHz). Then, by using Equation (7), the measured ECC at three points is noticed. The final response is drawn by curve fitting. It is generally desirable to have an ECC value below 0.5 for satisfactory MIMO performance [22,23]. Figure 13 illustrates the simulated and measured ECC of the presented MIMO antenna. The results clearly demonstrate that the ECC remains well below 0.1 in the frequency bands of interest. This low ECC value signifies excellent isolation between the antenna branches, which is essential for achieving high-quality MIMO communication.

(7) $$\rho_{e}=\frac{|\int_{0}^{2\pi }\int_{0}^{\pi }(XPR.E_{\theta 1}.E_{\theta 2}^{*}.P_{\theta }+E_{\varphi 1}.E_{\varphi 2}^{*}.P_{\varphi })d\Omega {|}^{2}}{\int_{0}^{2\pi }\int_{0}^{\pi }(XPR.E_{\theta 1}.E_{\theta 1}^{*}.P_{\theta }+E_{\varphi 1}.E_{\varphi 2}^{*}.P_{\varphi })d\Omega \times \int_{0}^{2\pi }\int_{0}^{\pi }(XPR.\;E_{\theta 2}.E_{\theta 2}^{*}.P_{\theta }+E_{\varphi 2}.E_{\varphi 2}^{*}.P_{\varphi })d\Omega }$$

### 6.2. Diversity Gain (DG)

**Figure 14:**
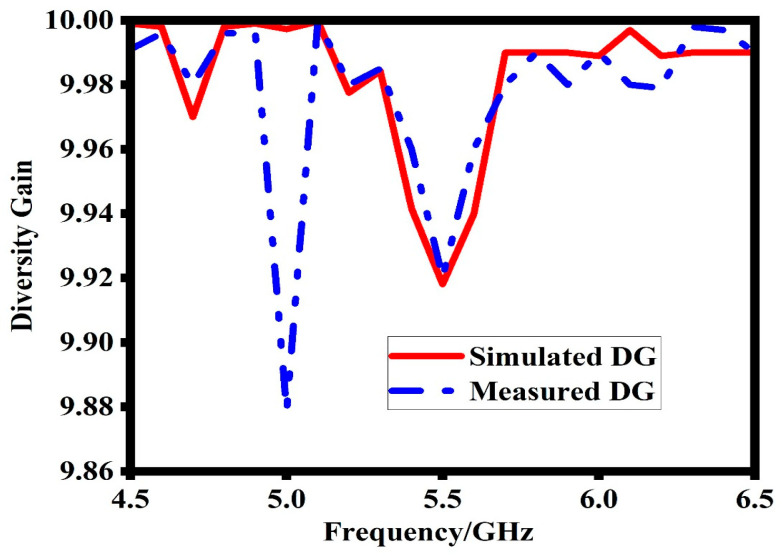
Simulated and measured diversity gain of the proposed design.

The diversity gain (DG) is another important parameter that evaluates the enhancement provided by a MIMO system compared to a single antenna system. The DG is calculated using Equation (8), which quantifies the improvement in signal quality achieved through diversity techniques. Figure 14 shows the simulated and measured DG of the proposed MIMO antenna. The results indicate that in the frequency bands of interest, the DG is approximately 10 dB. This high DG value signifies excellent MIMO performance for a two-element MIMO system [23]. The achieved DG highlights the effectiveness of the proposed antenna design in improving signal reliability and robustness in MIMO communication scenarios.

(8) $$DG=10\sqrt{\left(1-{ECC}^{2}\right)}$$

### 6.3. Channel Capacity Loss (CCL)

Channel capacity loss (CCL) is another important MIMO parameter. CCL is important in showcasing the effectiveness of the proposed design throughput. The smaller the value of the CCL, the better the data transmission. A 0.4 bits/s/Hz CCL value is considered a good one for good data transmission. Equations (9)–(12) explain the basic parameters which help to determine the final CCL response of the proposed design [23].

(9) $$C\left(Loss\right)=-\log_{2}det(\psi^{R})$$

where $\psi^{R}$ is the correlation matrix at the receiving antenna.

(10) $$\psi^{R}=\left[\begin{array}{cc} \rho 11 & \rho 12\\ \rho 21 & \rho 22 \end{array}\right]$$

(11) $$\rho \mathrm{ii}=1-\left({\left|Sii\right|}^{2}+{\left|Sij\right|}^{2}\right),\;\mathrm{for}\;i,\;j=1\mathrm{or}2$$

(12) $$\rho \mathrm{ii}=-\left(Sii*Sij+Sji*Sij\right),\;\mathrm{for}\;i,\;j=1\mathrm{or}2$$

**Figure 15:**
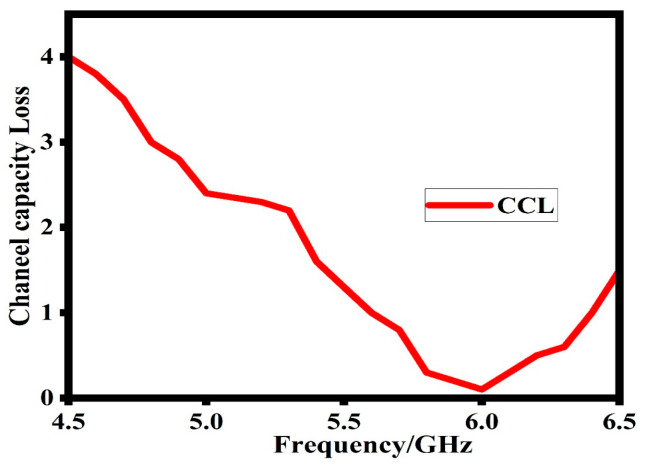
Simulated channel capacity loss (CCL) of the proposed design.

Figure 15 shows that at the target operating band, the proposed design has maintained less than 0.02 bits/s/Hz value of the CCL; this value of CCL shows a good data transmission and throughput.

### 6.4. Mean Effective Gain (MEG)

**Figure 16:**
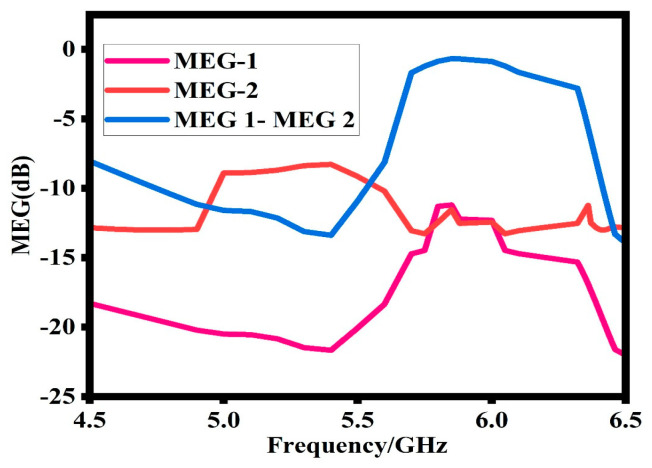
Simulated mean effective gain (MEG) of the proposed design.

Mean effective gain (MEG) is another important MIMO antenna design parameter. It is the ratio between the diversity antennas’ received power and the isotropic antenna’s received power [23]. It shows the effectiveness of the antenna to accept electromagnetic power in a multipath environment. The MEG is calculated based on Equations (13) and (14). For good performance, the MEG between the antenna ports should be less than 3 dB. Figure 16 shows that at the operating band of the proposed design, the value of the MEG is around 0 dB.

MEGi = 0.5[1 − |Sii|^2^ − |Sii|^2^] (13)

MEGi = 0.5[1 − |Sij|^2^ − |Sjj|^2^] (14)

### 6.5. Total Active Reflection Coefficient (TARC)

The total active reflection coefficient is also an important MIMO antenna parameter. It is the ratio between the square root of the total power reflected and the total power incident. It is helpful in the determination of the effective operating bandwidth of a MIMO antenna system. The TARC of a MIMO antenna system is determined with the help of the following equations [23]:

(15) $$\mathrm{TARC}=N^{-0.5}*\sqrt{\sum_{i=1}^{N}\left|\sum_{k=i}^{N}s_{{ik}^{e^{j\theta }k-1}}\right|2}$$

**Figure 17:**
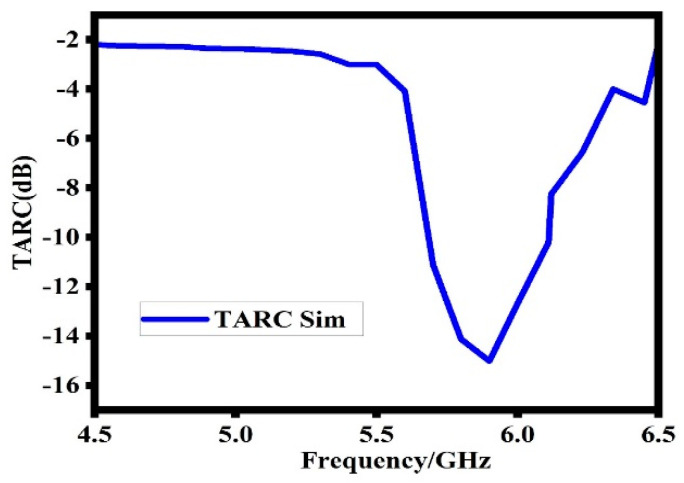
Simulated total active reflection coefficient of the proposed design.

For good communication, the TARC of a MIMO system should under 0 dB [24]. Thus, any value of the TARC under 0 dB is considered a good one for better communication. Figure 17 shows that the proposed design has less than 13 dB TARC at the operating band. This value of TARC is considered a better one for the proposed design.

A comprehensive comparison between the proposed work and recently published studies is presented in Table 2. The comparison focuses on several key parameters, including electrical and physical size, type of antennas, maximum gain, and isolation between the MIMO elements. From the comparison, it is evident that the proposed work offers a compact size while delivering impressive performance characteristics. The antenna design has a smaller electrical and physical size compared to the published studies, indicating its potential for space-constrained applications. Moreover, the isolation achieved through the novel decoupling technique is high, while keeping the design compact. This confirms the suitability of the proposed design for real-time wireless applications.

## 7. Conclusions

Mutual coupling between closely placed dielectric resonator antennas on a multiple antenna system was investigated in the present work. A single-negative magnetic (MNG) H-SRR band gap decoupling structure was examined. Detailed simulation and experimental measurements were performed as well. The magnetic insertion has shown the effectiveness in terms of isolation enhancement and suppressing the displacement current. From the simulation results, a 6 dB isolation was achieved without H-SRR insertion and 27 dB additional isolation was achieved at the resonance frequency by employing the H-SRR. The isolation enhancement has resulted in a recovery of the multiple antennas pattern, which is clear from the pattern measurement performance. The computed results confirm that implementing the H-SRRs between the antennas offers an excellent coupling reduction. The benefit of the proposed design is its small electrical size (λ/12) and simplicity of fabrication as compared to other meta-structures. The overall design compactness and novel decoupling structure emphasizes the significance of the proposed design for real-time wireless applications.

## Figures and Tables

**Table 1:** Dimensions table of proposed MMO-DRA.

Parameters	Dimensions (mm)	Parameters	Dimensions (mm)
A, b	13	L_s_	40
d	3.15	h_s_	1.6
W_s_	58.3	D_1_	λ/4
R, g	3.9, 0.5	c	0.6

**Table 2:** Comparison of the proposed design with the state of the art.

Ref.	Dimension (mm^2^) /λg^2^	Type of Antenna	Gain (dBi)	Isolation (dB)
[23]	60 × 60 × 10.054 (0.8λ × 0.8λ × 0.13λ)	Fractal DRA	Not mentioned	~15
[25]	30 × 30 × 8.1 (1.85λ × 1.85λ × 0.145λ)	Printed	5	18
[26]	100 × 100 × 8.14 (0.79λ × 0.79λ × 0.1)	CDRA	2.1	20
[27]	50 × 50 × 14.6 (1.93λ × 1.93λ × 0.15λ)	CDRA	Not mentioned	25
[28]	160 × 160 × 14 (3.1λ × 3.1λ × 0.14λ)	CDRA	Not mentioned	15
[29]	56.6 × 31.5 × 14.09 (1.69λ × 0.94λ × 0.27λ)	RDRA	Not mentioned	20
[30]	78 × 50 × 6.4 (1.71λ × 2.67λ × 0.057λ)	CDRA	2.3	20
[24]	70 × 60x9.1 (1.12λ × 0.96λ × 0.14λ)	RDRA	Not mentioned	12
[31]	40 × 65 × 7.6 (1.44λ × 0.88λ)	RDRA	4	19
[32]	46 × 46 × 21.6 (2.17λ × 2.17λ × 0.216)	RDRA	Not mentioned	24
Proposed	40 × 58.3 × 4.75 (1.5λ × 1.02λ × 0.079)	RDRA	5	30

## Data Availability

The data will be available on request.

## References

[1] Capolino F. (2017). Applications of Metamaterials.

[2] Eleftheriades G.V., Balmain K.G. (2005). Negative-Refraction Metamaterials: Fundamental Principles and Applications.

[3] Salicrú Cortés M. (2009). Study of Performance of Reference MIMO Antenna Configurations using Experimental Propagation Data. Master’s Thesis.

[4] Abdullah S., Xiao G., Amaya R.E. (2021). A review on the history and current literature of metamaterials and its applications to antennas & radio frequency identification (RFID) devices. IEEE J. Radio Freq. Identif..

[5] Saadh A.M., Ashwath K., Ramaswamy P., Ali T., Anguera J. (2020). A uniquely shaped MIMO antenna on FR4 material to enhance isolation and bandwidth for wireless applications. AEU-Int. J. Electron. Commun..

[6] Kulkarni J., Alharbi A.G., Elfergani I., Anguera J., Zebiri C., Rodriguez J. (2022). Dual polarized, multiband four-port decagon shaped flexible MIMO antenna for next generation wireless applications. IEEE Access.

[7] Nej S., Ghosh A., Ahmad S., Ghaffar A., Hussein M. (2022). Compact quad band MIMO antenna design with enhanced gain for wireless communications. Sensors.

[8] Yang F., Rahmat-Samii Y. (2003). Microstrip antennas integrated with electromagnetic band-gap (EBG) structures: A low mutual coupling design for array applications. IEEE Trans. Antennas Propag..

[9] Ghosh S., Tran T.N., Le-Ngoc T. (2014). Dual-layer EBG-based miniaturized multi-element antenna for MIMO systems. IEEE Trans. Antennas Propag..

[10] Xin H., Matsugatani K., Kim M., Hacker J., Higgins J.A., Rosker M., Tanaka M. (2002). Mutual coupling reduction of low-profile monopole antennas on high-impedance ground plane. Electron. Lett..

[11] Gheethan A.A., Herzig P.A., Mumcu G. (2013). Compact 2 × 2 coupled double loop GPS antenna array loaded with broadside coupled split ring resonators. IEEE Trans. Antennas Propag..

[12] Qamar Z., Riaz L., Chongcheawchamnan M., Khan S.A., Shafique M.F. (2014). Slot combined complementary split ring resonators for mutual coupling suppression in microstrip phased arrays. IET Microw. Antennas Propag..

[13] Bait-Suwailam M.M., Boybay M.S., Ramahi O.M. (2010). Electromagnetic coupling reduction in high-profile monopole antennas using single-negative magnetic metamaterials for MIMO applications. IEEE Trans. Antennas Propag..

[14] Wiltshire MC K., Hajnal J.V., Pendry J.B., Edwards D.J., Stevens C.J. (2003). Metamaterial endoscope for magnetic field transfer: Near field imaging with magnetic wires. Opt. Express.

[15] Roshna T.K., Deepak U., Sajitha V.R., Vasudevan K., Mohanan P. (2015). A compact UWB MIMO antenna with reflector to enhance isolation. IEEE Trans. Antennas Propag..

[16] Yeom I., Kim H., Jung J., Jung C.W. (2015). Analysis of spatial/polarization diversity using a broadband slot-coupled patch antenna for the WLAN 802.11 A/B/G/N access point. Microw. Opt. Technol. Lett..

[17] Baena J.D., Marqués R., Medina F., Martel J. (2004). Artificial magnetic metamaterial design by using spiral resonators. Phys. Rev. B.

[18] Selvaraju R., Jamaluddin M.H., Kamarudin M.R., Nasir J., Dahri M.H. (2018). Complementary split ring resonator for isolation enhancement in 5G communication antenna array. Prog. Electromagn. Res. C.

[19] Shamonin M., Shamonina E., Kalinin V., Solymar L. (2004). Properties of a metamaterial element: Analytical solutions and numerical simulations for a singly split double ring. J. Appl. Phys..

[20] Sauviac B., Simovski C.R., Tretyakov S.A. (2004). Double split-ring resonators: Analytical modeling and numerical simulations. Electromagnetics.

[21] Chen L., Ma Q., Luo S.S., Ye F.J., Cui H.Y., Cui T.J. (2022). Touch-Programmable Metasurface for Various Electromagnetic Manipulations and Encryptions. Small.

[22] Ma Q., Gao W., Xiao Q., Ding L., Gao T., Zhou Y., Gao X., Yan T., Liu C., Gu Z. (2022). Directly wireless communication of human minds via non-invasive brain-computer-metasurface platform. Elight.

[23] Trivedi K., Pujara D. (2017). Suppression of mutual coupling in wideband tree shaped fractal DRA array for MIMO applications. Proceedings of the 2017 11th European Conference on Antennas and Propagation (EUCAP).

[24] Sahu N.K., Gangwar R.K., Kumari P. (2018). Dielectric resonator based circularly polarized MIMO antenna for WLAN applications. Proceedings of the 2018 3rd International Conference on Microwave and Photonics (ICMAP).

[25] Das G., Sharma A., Gangwar R.K., Sharawi M.S. (2018). Compact back-to-back DRA-based four-port MIMO antenna system with bi-directional diversity. Electron. Lett..

[26] Zou L., Abbott D., Fumeaux C. (2012). Omnidirectional cylindrical dielectric resonator antenna with dual polarization. IEEE Antennas Wirel. Propag. Lett..

[27] Das G., Sharma A., Gangwar R.K. (2017). Dual port aperture coupled MIMO cylindrical dielectric resonator antenna with high isolation for WiMAX application. Int. J. RF Microw. Comput.-Aided Eng..

[28] Sharawi M.S., Podilchak S.K., Khan M.U., Antar Y.M. (2017). Dual-frequency DRA-based MIMO antenna system for wireless access points. IET Microw. Antennas Propag..

[29] Abdalrazik A., Abd El-Hameed A.S., Abdel-Rahman A.B. (2017). A three-port MIMO dielectric resonator antenna using decoupled modes. IEEE Antennas Wirel. Propag. Lett..

[30] Sharma A., Das G., Gangwar R.K. (2016). Dual polarized triple band hybrid MIMO cylindrical dielectric resonator antenna for LTE2500/WLAN/WiMAX applications. Int. J. RF Microw. Comput. -Aided Eng..

[31] Sahu N.K., Das G., Gangwar R.K. (2018). L-shaped dielectric resonator based circularly polarized multi-input-multi-output (MIMO) antenna for wireless local area network (WLAN) applications. Int. J. RF Microw. Comput.-Aided Eng..

[32] Chen H.N., Song J.M., Park J.D. (2019). A compact circularly polarized MIMO dielectric resonator antenna over electromagnetic band-gap surface for 5G applications. IEEE Access.

